# The Influence of the Explant’s Type on the Performance of Synthetic Seeds of Blackberry (*Rubus* spp.)

**DOI:** 10.3390/plants13010032

**Published:** 2023-12-21

**Authors:** Luca Regni, Maurizio Micheli, Simona Lucia Facchin, Alberto Marco Del Pino, Cristian Silvestri, Primo Proietti

**Affiliations:** 1 Department of Agricultural, Food and Environmental Sciences, University of Perugia, Borgo XX Giugno, 06121 Perugia, Italy; simonafacchin88@gmail.com (S.L.F.); alberto.delpino@unipg.it (A.M.D.P.); primo.proietti@unipg.it (P.P.); 2 Department of Agriculture and Forest Sciences, University of Tuscia, Via San Camillo de Lellis, 01100 Viterbo, Italy; silvestri.c@unitus.it

**Keywords:** small fruit, micropropagation, synseeds, vegetative propagules, alginate beads

## Abstract

In vitro propagation, also known as micropropagation, has become the most widely employed method for blackberry propagation, as it overcomes the limitations of the traditional asexual propagation methods (mainly layering and cutting). In this context, synthetic seed technology represents a strategy to enhance the productivity of in vitro propagation and facilitates the exchange of plant materials between laboratories, contributing to germplasm conservation. This study aimed to identify the most suitable vegetative propagule for the encapsulation of blackberry. To this end, uninodal microcuttings (nodes) and the base of clumps were used to produce synthetic seeds for the cultivars Thornfree and Chester. Forty-five days after sowing, viability (percentage of green propagules without browning or necrosis), regeneration (percentage of propagules that sprouted and rooted simultaneously), number of shoots produced, shoot length, number of roots produced, root length, and the fresh and dry weights of the plantlets were measured. The results demonstrated that both considered propagules allowed us to obtain satisfactory regeneration rates. However, plantlets originating from the encapsulated clump’s base had more shoots and roots, resulting in greater fresh and dry weights than the plantlets derived from encapsulated nodes. Therefore, for achieving more robust plantlets and enhancing overall procedural efficiency, we recommend using the base of clumps as a propagule for blackberry encapsulation.

## 1. Introduction

In recent years, there has been a consistent increase in the cultivation of blackberries and the consumption of fresh and processed blackberry fruits [1,2]. The rising blackberries’ consumption can be attributed to their appealing shapes, textures, flavors, and colors [3]. Moreover, the presence of bioactive compounds including flavonoids, cyanogenic glucosides, phytoestrogens, and phenols, coupled with their nutritional value, contributes to their positive effects on human diet and health [4]. Additionally, blackberry leaves, often regarded as a by-product of cultivation, represent an alternative source of bioactive compounds that can be used for the development of functional food products and nutraceuticals [5].

The Food and Agriculture Organization Corporate Statistical Database (FAOSTAT) does not report data about blackberry cultivation and consumption since they are included in the category ‘other berries’. However, a global survey conducted in 2005 reported that blackberry production occurs mainly in North America (59,123 tonnes) and Europe (43,000 tonnes) [6]. These numbers are expected to have risen as the blackberry industry has expanded significantly in recent years. This growth has been driven by increased consumption, the introduction of new cultivars, and the adoption of advanced production methods that enable a year-round yield. Notably, the fresh-market blackberry industry has seen rapid growth in Mexico, as well as in Southern Europe, Australia, and Central and South America [7].

Blackberry plants are successfully propagated by traditional methods such as layering and cuttings [8], ensuring the preservation of desirable agronomic characteristics [2,9]. However, these methods require a large plantation area, high labor demand, and intensive weed control [10]. To overcome the abovementioned limitations, the in vitro propagation (micropropagation) of blackberries has been successfully applied, and it now stands as the most common propagation method [9,10,11,12,13].

Successful micropropagation protocols have been developed for various blackberry cultivars including Marion, Black Satin, Thornless Evergreen, Loch Ness, Cacanska bestrna, Agavam, Ebano, Tupi, and Guarany [12,14,15]. In vitro methods were also used to study the effects of gamma irradiation on morphological and biochemical traits in blackberry plantlets [16]. However, it is important to underline that *Rubus* is a very wide and diverse genus, and the response of its species, hybrids, and cultivars to micropropagation is quite variable [9]. Extensive efforts are necessary to optimize the medium composition (nutrients, plant growth regulators, and their combinations) [4].

Furthermore, micropropagated plants are not easy to manage, store, and transport due to the risks of deterioration and damage. This can lead to commercial limitations when compared with the zygotic or gamic seeds, which, due to their reduced size, are easier to handle, store, and transport [17].

In this context, encapsulation is a promising technique that combines the advantages of micropropagation with some useful characteristics (e.g., reduced size) of zygotic seeds [17,18]. The technology of synthetic seeds ensures the genetic uniformity of plants, facilitates plant material handling and exchange between laboratories [19], and can be used for the short- to medium- and long-term conservation of germplasm [19] as well as for the eradication of viruses in several species of economic importance [20,21]. Initially, the definition of synthetic seeds was limited to encapsulated somatic embryos. However, given the recalcitrance of some plant species to produce somatic embryos, the concept of synthetic seeds was extended to a wide range of encapsulated vitro-derived propagules, including shoot tips, nodal segments, and others [19,22]. While numerous studies have explored the application of encapsulation technology in various plant species [19,23], this technology has rarely been applied to blackberry. Indeed, to our knowledge, only two studies have been published on synthetic seed technology in blackberry. One study focused on producing artificial seeds and inducing organogenesis [24], while another study aimed to develop a vitrification protocol for cryopreservation [25].

This study aimed to identify the most suitable vegetative propagule for the encapsulation of Thornfree and Chester blackberry cultivars. In particular, for the first time, to the best of our knowledge, the use of the clump’s base was evaluated and compared to uninodal microcutting as a vegetative propagule in blackberry encapsulation.

## 2. Results

In this study, we investigated the effect of explant type and explant age on the development of the synthetic seeds of two blackberry cultivars. The ANOVA results (Supplementary Table S1) did not show a significant interaction between these two factors or a significant effect of the explant age on any of the growth parameters investigated, except the number of roots formed by Chester explants (Supplementary Table S1, Table 1). In Chester, encapsulated explants that had been grown for 45 days before encapsulation formed significantly more roots (averaging 4.06 ± 0.64) than when the explants had been grown for 30 days (averaging 2.33 ± 0.18). Anyway, as the number of roots was enough to allow for a successful acclimation, we decided to pool the data of the two explant ages and focus on the effect of the explant type, which had a significant effect on the performance of both cultivars (Table 1).

**Figure 1:**
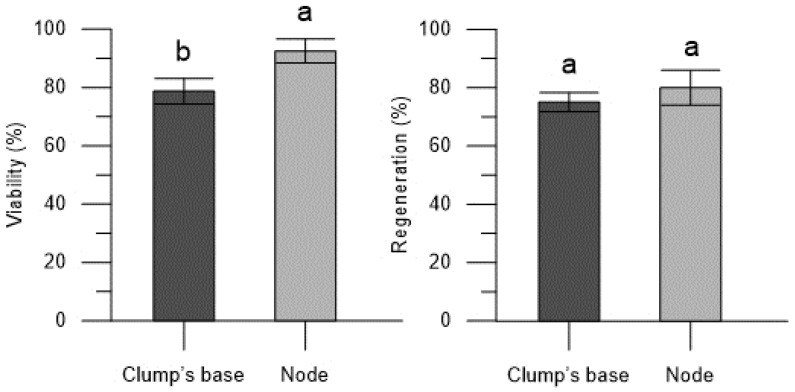
Viability and regeneration rates of the encapsulated explants (clump’s base and node) of blackberry Thornfree cultivar. Data are expressed as means ± SEM from 4 independent tests. Different letters indicate statistically significant differences according to Tukey HSD test (*p* ≤ 0.05).

In the Thornfree cultivar, the propagule type had an impact on viability, with a higher viability observed in the encapsulated nodes (92.50 ± 4.12%) compared to the clump’s base (78.75 ± 4.41%) (Figure 1). Nevertheless, it is worth noting that the encapsulated clump’s base exhibited a high and satisfactory viability rate. In contrast, regeneration rates were consistent and were not influenced by the type of explant used for encapsulation (Figure 1).

**Figure 2:**
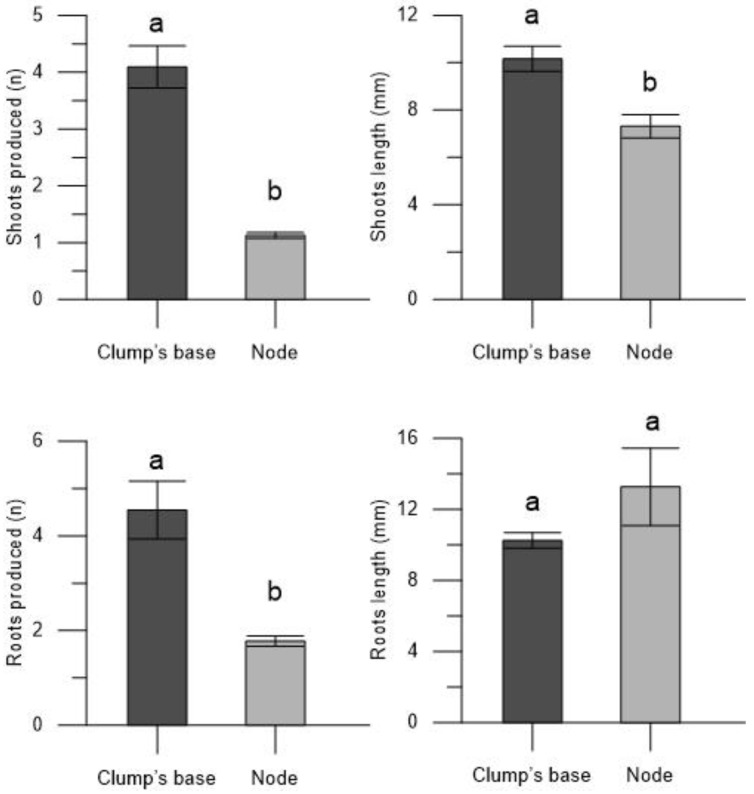
Number of shoots, shoots length, number of roots, and roots length in the plantlets derived from encapsulated clump’s base and node of blackberry Thornfree cultivar. Data are expressed as means ± SEM from 4 independent tests. Different letters indicate statistically significant differences according to Tukey HSD test (*p* ≤ 0.05).

The plantlets derived from the encapsulated clump’s base in the Thornfree cultivar exhibited a greater number of shoots (4.09 ± 0.37) and longer shoot lengths (10.16 ± 0.53 mm) compared to those derived from encapsulated nodes (1.13 ± 0.05 and 7.31 ± 0.49 mm, respectively) (Figure 2). Also, the number of roots was higher in the plantlets derived from the encapsulated clump’s bases (4.55 ± 0.61) than those derived from the encapsulated nodes (1.77 ± 0.11). However, it is noteworthy that root length was not influenced by the propagule type; root length remained consistent and was similar in plantlets derived from both the encapsulated nodes and clump’s base (Figure 2).

**Figure 3:**
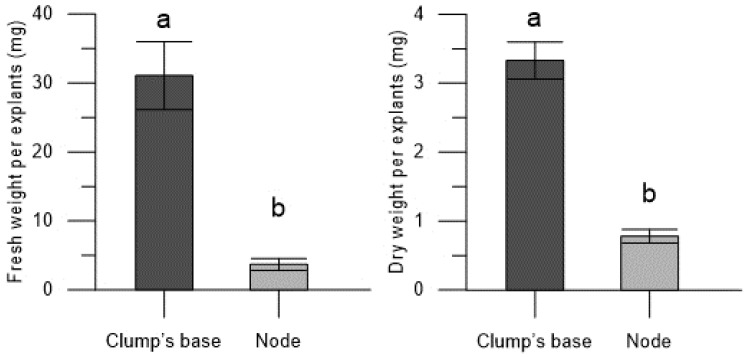
Fresh and dry weights of the plantlets derived from encapsulated clump’s base and node of blackberry Thornfree cultivar. Data are expressed as means ± SEM from 4 independent tests. Different letters indicate statistically significant differences according to Tukey HSD test (*p* ≤ 0.05).

**Figure 4:**
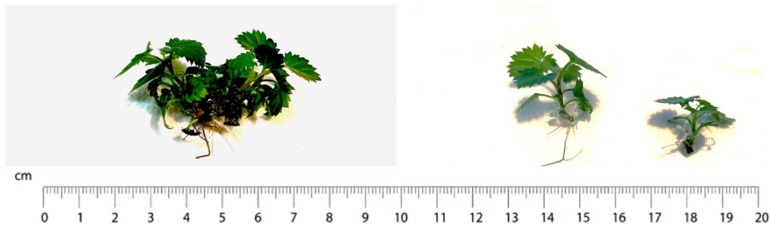
Plantlets of Thornfree cultivar obtained from encapsulated clump’s base on the (**left**) and encapsulated uninodal microcutting (node) on the (**right**).

The enhanced growth of Thornfree cultivar plantlets, derived from the encapsulated clump’s base, is evident in terms of shoots number, shoots length, and roots number. This is further supported by the higher fresh (31.07 ± 4.93 mg for the encapsulated clump’s base and 3.09 ± 0.87 mg for encapsulated nodes) and dry weights (3.33 ± 0.27 mg for the encapsulated clump’s base and 0.78 ± 0.10 mg for encapsulated nodes) of the plantlets (Figure 3 and Figure 4).

**Figure 5:**
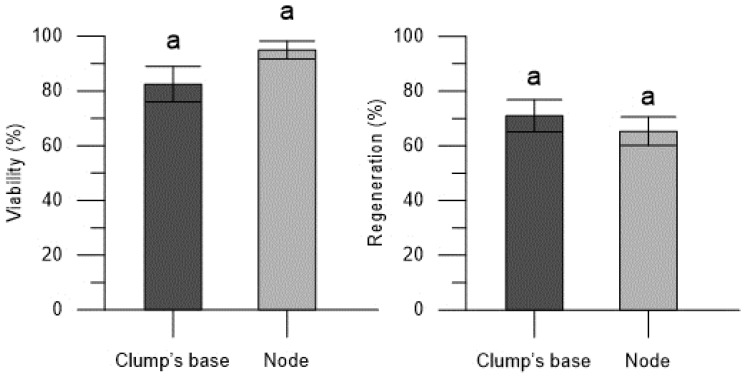
Viability and regeneration rates of the encapsulated explants (clump’s base and node) of blackberry Chester cultivar. Data are expressed as means ± SEM from 4 independent tests. Different letters indicate statistically significant differences according to Tukey HSD test (*p* ≤ 0.05).

The explant type used for encapsulation for the Chester cultivar did not affect the viability rate (Figure 5). Likewise, the regeneration rate of the encapsulated explants in the Chester cultivar was not affected by the type of propagule (Figure 5) as already observed for the Thornfree cultivar.

**Figure 6:**
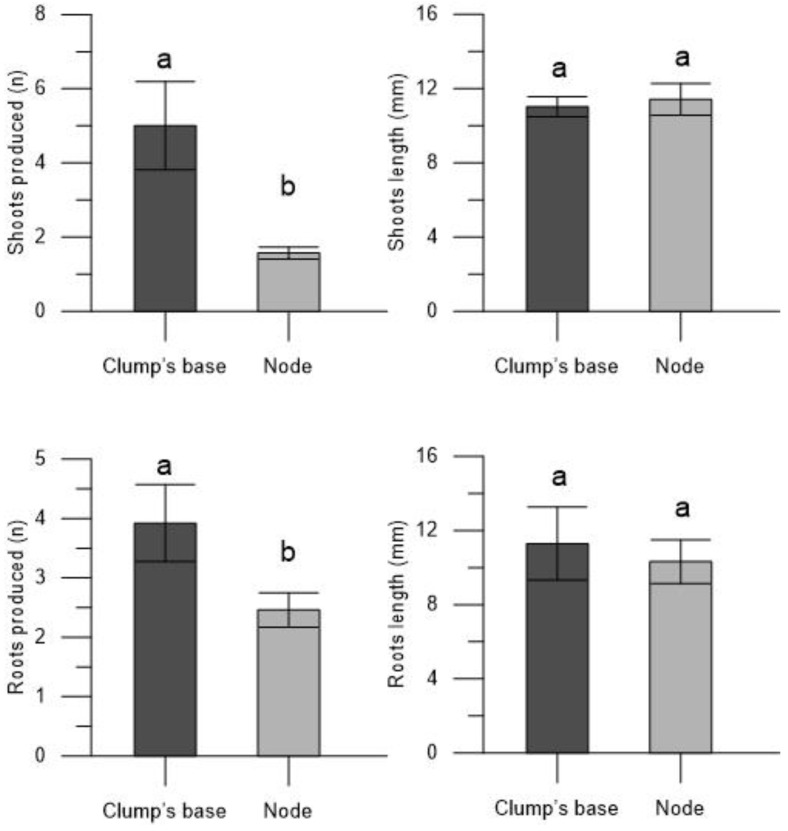
Number of shoots, shoots length, number of roots, and roots length in the plantlets derived from encapsulated clump’s base and node of blackberry Chester cultivar. Data are expressed as means ± SEM from 4 independent tests. Different letters indicate statistically significant differences according to Tukey HSD test (*p* ≤ 0.05).

Regarding the development of plantlets derived from encapsulated clump bases and nodes, differences were found only for the parameters number of shoots and number of roots (Figure 6). Specifically, the plantlets originating from the encapsulated clump base exhibited a higher number of shoots (5.00 ± 1.19) and roots (3.29 ± 0.65) compared to those originating from the encapsulated nodes (1.57 ± 0.16 and 2.46 ± 0.29 for number of shoots and roots, respectively). In contrast, the type of propagule used did not affect the length of shoots and roots (Figure 6).

**Figure 7:**
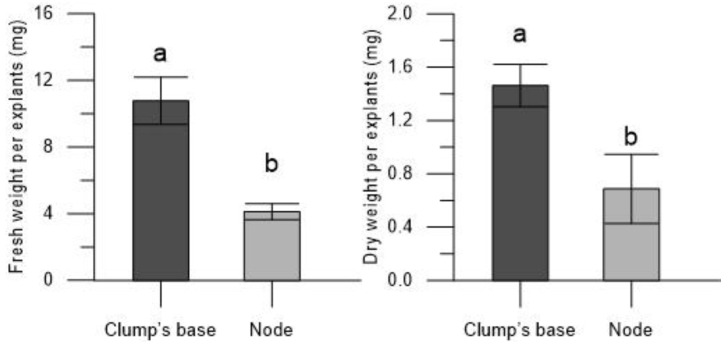
Fresh and dry weights of the plantlets derived from encapsulated clump’s base and node of blackberry Chester cultivar. Data are expressed as means ± SEM from 4 independent tests. Different letters indicate statistically significant differences according to Tukey HSD test (*p* ≤ 0.05).

**Figure 8:**
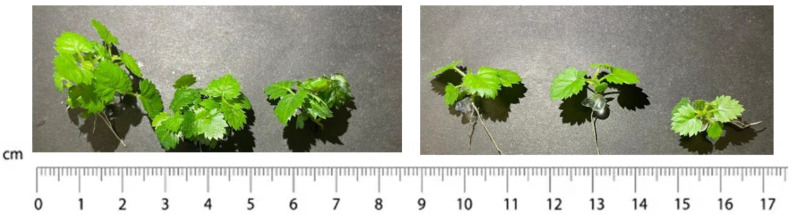
Plantlets of Chester cultivar obtained from encapsulated clump’s base on the (**left**) and encapsulated uninodal microcutting (node) on the (**right**).

The plantlets originating from the encapsulated clump’s base exhibited a greater fresh weight (10.77 ± 1.42 mg) and dry weight (1.46 ± 0.16) compared to those derived from encapsulated nodes (4.12 ± 0.48 mg for fresh weight and 0.69 ± 0.99 mg for dry weight) (Figure 7 and Figure 8), and this can be attributed to the higher number of shoots observed.

## 3. Discussion

In the present study, the possibility of using the in vitro-derived base of the clump as a propagule for blackberry encapsulation was investigated for the first time. The type of propagule along with the composition of the artificial endosperm, the sowing medium, and the growing conditions is a factor that can strongly influence the performance of the synthetic seeds [19].

Both types of evaluated propagules provided positive results in terms of viability and regeneration rates, demonstrating that they can absorb the needed water and nutrients from the capsule. The high regeneration rates obtained are significant given the consensus among researchers regarding the crucial importance of obtaining a high regeneration rate. Indeed, only in this case, the synthetic seed technology is truly valuable and applicable [26]. Nodes with apical or axillary buds (microcuttings) are widely used for synthetic seed production since they can be obtained easily and have a higher degree of genetic stability compared to somatic embryos [27,28]. For this reason, these vegetative propagules have been used to obtain synthetic seeds in several plant species including pineapple [29], Carrizo citrange [30], pomegranate [31], kiwifruit [32], apple rootstock M26 [27,28,33], mulberry [34,35], olive [26,36], and hop [37].

However, one potential drawback of using microcuttings for encapsulation may arise from the absence of root primordia, thereby impeding the spontaneous formation of adventitious roots. However, in our study, the regeneration rates obtained were satisfactory with both vegetative propagules (nodes and clump’s bases) encapsulated in Thornfree and Chester cultivars. In particular, for the Thornfree cultivar, the regeneration rates exceeded 80%. This result is consistent with what has been observed in other species, such as banana [38], *Cannabis* [39], *Dalbergia* [40], *Plumbago* [41], and *Solanum nigrum* [42], where the encapsulated microcuttings exhibited a high rooting capacity after sowing. On the contrary, in other species such as *Borivilianum* [43], olive [36], *Phyllanthus* [44], pomegranate [31], *Spilanthes* [45], *Tylophora* [46], and several others [19], rooting was not satisfactory.

Among the two propagules examined in this study, the clump’s base seems to be the more suitable for encapsulation as it gives rise, after regeneration, to plantlets with a higher number of shoots and roots in both cultivars. This could be attributed to the abundance of meristematic cells present in the clump’s base [47]. Explant age does not appear to be a critical factor for synthetic seed development. This flexibility can be an advantage for carrying out experiments with a high number of propagules or for the transfer of this technique to large-scale propagation companies.

## 4. Materials and Methods

### 4.1. Plant Material

In vitro-derived material of blackberry cultivars Thornfree and Chester was obtained from the experimental fields of the ‘Tree Science’ Research Unit of the Department of Agricultural, Food and Environmental Sciences, and used as the object of the study.

Thornfree is a very vigorous cultivar with stems that can reach up to 5 m in length and up to 2 m in height. The flowers are large and white (with a low pinkish shade). The Thornfree cultivar has a late ripening and a long harvest season. The fruits are large, black, and glossy with an oblong shape. The fruit’s firmness is very low because Thornfree’s berries have a high juicy content. Thornfree cultivar has a medium resistance to pests and disease and a good resistance to low temperatures. On the contrary, Thornfree cultivar’s berries are sensitive to high summer temperatures that cause sunburns, so they need to be sheltered from direct sun rays [48].

The Chester cultivar is a semi-erect shrub characterized by high yield potential and tolerance to pests and diseases. The fruits are big, characterized by good quality, late maturation, and good postharvest performance. The plant has a semi-erect growth habit and needs a supporting system [49].

### 4.2. Encapsulating Procedure and Growing Conditions

For the proliferation of the Thornfree and Chester cultivars, glass jars (500 mL) were used, each containing 100 mL of a growth medium consisting of the half-strength nutrient component of the Murashige and Skoog medium [50] supplemented with sucrose (15 g L^−1^), Indole-3-butyric acid (IBA) (0.1 mg L^−1^), 6-Benzylaminopurine (BAP) (0.4 mg L^−1^), and agar (8 gL^−1^), pH of 5.7. The vessels were placed in a growth chamber at a constant temperature of 22 ± 2 °C and a 16 h photoperiod of light with an intensity of 40 µE m^−2^ s^−1^. The usual proliferation subculture length was 30 days.

**Figure 9:**
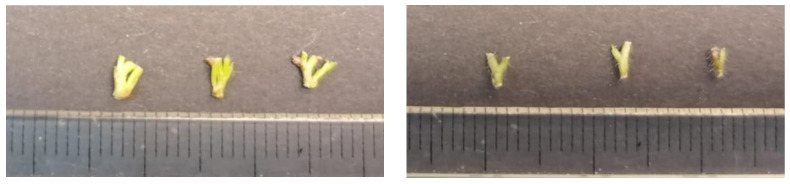
Explants used for encapsulation: clump’s base on the (**left**) and uninodal microcuttings (nodes) on the (**right**).

Shoots (about 4 cm length) were selected from a well-established in vitro plant material (four proliferation subcultures) from plant material derived from two subcultures of different durations (30 and 45 days). From the abovementioned shoots, uninodal microcuttings (3–4 mm long, hereafter referred to as nodes) and the clump’s bases (3–4 mm long) were isolated and encapsulated to verify which was the most suitable (Figure 9).

**Figure 10:**
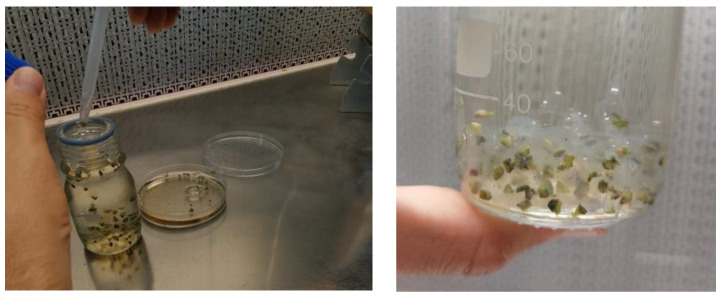
Propagules with a drop of the coating solution were dropped in the complexing solution (**left**) and encapsulated explant in the complexing solution (**right**).

Encapsulation was performed according to the procedure described by Standardi and Micheli [17]. The explants were individually collected and dropped for 15 min into a coating solution consisting of the one-quarter-strength nutrient component of the Murashige and Skoog medium [50] supplemented with sucrose (50 g L^−1^), IBA (0.1 mg L^−1^), BAP (0.4 mg L^−1^), and sodium alginate (25 g L^−1^) of medium viscosity (Sigma Aldrich), and deprived of calcium chloride, with a pH of 5.7. The explants were then taken with a drop of coating solution using sterile Pasteur pipettes and individually collected and dropped for 30 min into the complexing solution consisting of the one-quarter-strength nutrient component of the Murashige and Skoog medium [50] supplemented with sucrose (50 g L^−1^), IBA (0.1 mg L^−1^), BAP (0.4 mg L^−1^), and calcium chloride (11 g L^−1^) (Merck KGaA, Darmstadt, Germany), pH of 5.7 (Figure 10).

The encapsulated explants were then taken with the aid of sterile forceps and rinsed with a sterile washing solution consisting of the one-quarter-strength nutrient component of the Murashige and Skoog medium [50] supplemented with sucrose (50 g L^−1^), IBA (0.1 mg L^−1^), and BAP (0.4 mg L^−1^), with a pH of 5.7; this was performed three times for 15 min to remove the eventual presence of toxic residual ions of chloride and sodium.

The capsules (diameter 5–6 mm) were sown, respecting polarity in glass jars (500 mL volume) containing 100 mL of the growth medium consisting of the Murashige and Skoog medium [50] supplemented with sucrose (30 g L^−1^) and agar (8 g L^−1^), with a pH of 5.7. In each jar, 10 capsules of propagules were sown, and 4 replicates for each treatment were set up. The procedure described above took place in sterile conditions under a laminar flow hood at room temperature (about 23 °C).

The jars containing the encapsulated propagules were placed in a growth chamber at a constant temperature of 22 ± 2 °C and a 16 h photoperiod of light with an intensity of 40 µE m^−2^ s^−1^. All the plant’s manipulations were carried out in sterile conditions under horizontal laminar flow cabinet.

### 4.3. Growth Parameters

The evaluation of the following parameters was made 45 days after sowing: viability (green propagules without browning or necrosis) refers to the initial explants (%), regeneration (propagules that sprouted and rooted at the same time) refers to the initial explants (%), average number of shoots produced per explant that showed shoots and roots (n), average shoots length per explant that showed shoots and roots (mm), average roots produced per explant that showed shoots and roots (n), average roots length per explant that showed shoots and roots (mm), average fresh weight per explant that showed shoots and roots (mg), and average dry weight per explant that showed shoots and roots (mg) obtained by keeping the plant material in an oven for three days at 105 °C.

### 4.4. Statistical Analysis

The trial was organized according to a completely randomized design. For each cultivar, two types of explant (node and clump’s base) and two explant ages (30 and 45 days) were considered, with 40 encapsulated explants for each treatment. The collected data were subjected to a two-way ANOVA analysis (Split-Plot completely randomized design), and the significance of the differences was tested using the Tukey HSD test (*p* < 0.05). Data on percentages were arcsine-transformed before performing statistical analysis.

## 5. Conclusions

Both vegetative propagules used in this study (uninodal microcutting and clump’s base) are suitable for encapsulation since they exhibited a high regeneration rate. The base of the clumps due to the abundance of meristematic tissue gives rise to plantlets with a higher number of shoots and roots than unimodal microcuttings in both cultivars. Despite their great vegetative vigor, the bases of the clumps are rarely used for in vitro multiplication. Nowadays, individual shoots are mainly used for in vitro propagation, but in the future, clump bases could be isolated with automated systems to separate them from the proliferated biomass. Indeed, automation is another possibility for reducing the cost of in vitro-derived production, but the systems studied so far cannot accurately separate individual shoots, often damaging them mechanically. Instead, the base of the clump, which can be evenly fragmented owing to its structure, appears to be capable of developing new shoots and plantlets after encapsulation. The findings from the present study represent the first evidence wherein the base of the clumps in blackberry cultivars Thornfree and Chester was identified as the optimal propagule for encapsulation.

## Figures and Tables

**Table 1:** Summary of the ANOVA results of the effects of the explant type and explant age factors and their interaction on the viability (%), regeneration (%), shoot produced (n), shoot length (mm), roots produced (n), roots length (mm), fresh weight per explant (mg), and dry weight per explant (mg) for Thornfree and Chester cultivars. The complete ANOVA tables for each parameter can be found in Supplementary Table S1.

Factors	Viability (%)	Regeneration (%)	Shoot Produced (n)	Shoot Length (mm)	Roots Produced (n)	Roots Length (mm)	Fresh Weight per Explant (mg)	Dry Weight per Explant (mg)
**Cultivar Thornfree**								
Type of explant	*	ns	**	**	**	ns	**	**
Explant age	ns	ns	ns	ns	ns	ns	ns	ns
Interaction	ns	ns	ns	ns	ns	ns	ns	ns
**Cultivar Chester**								
Type of explant	ns	ns	*	ns	*	ns	**	**
Explant age	ns	ns	ns	ns	*	ns	ns	ns
Interaction	ns	ns	ns	ns	ns	ns	ns	ns

* *p* ≤ 0.05; ** *p* ≤ 0.01; ns, none significant.

## Data Availability

The data that support the findings of this study are available from the corresponding author upon reasonable request.

## Supplementary Materials

The following supporting information can be downloaded at: https://www.mdpi.com/article/10.3390/plants13010032/s1, Table S1: ANOVA tables.

## References

[1] Muñoz-Concha D., Quintero J., Ercişli S. (2021). Media and Hormones Influence in Micropropagation Success of Blackberry cv. ‘Chester’. Res. J. Biotechnol. Vol..

[2] Vujović T., Ružić D., Cerović R., Leposavić A., Karaklajić-Stajić Ž., Mitrović O., Žurawicz E. (2017). An Assessment of the Genetic Integrity of Micropropagated Raspberry and Blackberry Plants. Sci. Hortic..

[3] Hunková J., Gajdošová A., Szabóová M. (2020). Effect of Mesos Components (MgSO_4_, CaCl_2_, KH_2_PO_4_) on In Vitro Shoot Growth of Blackberry, Blueberry, and Saskatoon. Plants.

[4] Kefayeti N., Kafkas E., Ercişli S. (2019). Micropropagation of’Chester Thornless’ Blackberry Cultivar Using Axillary Bud Explants. Not. Bot. Horti Agrobot..

[5] Kolarević T., Milinčić D.D., Vujović T., Gašić U.M., Prokić L., Kostić A.Ž., Cerović R., Stanojevic S.P., Tešić Ž.L., Pešić M.B. (2021). Phenolic Compounds and Antioxidant Properties of Field-Grown and in Vitro Leaves, and Calluses in Blackberry and Blueberry. Horticulturae.

[6] Foster T.M., Bassil N.V., Dossett M., Leigh Worthington M., Graham J. (2019). Genetic and Genomic Resources for Rubus Breeding: A Roadmap for the Future. Hortic. Res..

[7] Hall H.K. (2017). World Blackberry Production. Blackberries and Their Hybrids.

[8] Zia-Ul-Haq M., Riaz M., De Feo V., Jaafar H.Z., Moga M. (2014). *Rubus Fruticosus* L.: Constituents, Biological Activities and Health Related Uses. Molecules.

[9] Reed B., Poothong S., Hall H.K. (2017). Propagation of Blackberries and Related *Rubus* Species. Blackberries and Their Hybrids.

[10] Gomes H.T., Bartos P.M.C., Andrade M.T.D., Almeida R.F., Lacerda L.F.D., Scherwinski-Pereira J.E. (2017). In Vitro Conservation of Blackberry Genotypes under Minimal Growth Conditions and Subsequent Large-Scale Micropropagation. Pesqui. Agropecu. Bras..

[11] Ayub R.A., Santos J.N.D., Zanlorensi Junior L.A., Silva D.M.D., Carvalho T.C.D., Grimaldi F. (2019). Sucrose Concentration and Volume of Liquid Medium on the In Vitro Growth and Development of Blackberry cv. Tupy in Temporary Immersion Systems. Ciênc. Agrotecnol..

[12] Bobrowski V.L., Mello-Farias P., Petters J. (1996). Micropropagation of Blackberries (*Rubus* Sp.) Cultivars. Curr. Agric. Sci. Technol..

[13] Najaf-Abadi A.J., Hamidoghli Y. (2009). Micropropagation of Thornless Trailing Blackberry (‘*Rubus* sp.’) by Axillary Bud Explants. Aust. J. Crop Sci..

[14] Ružić D., Lazić T. (2006). Micropropagation as Means of Rapid Multiplication of Newly Developed Blackberry and Black Currant Cultivars. Agric. Conspec. Sci..

[15] Lepse L., Laugale V. Micropropagation, Rooting and Acclimatization of Blackberry ‘Agavam’. Proceedings of the I International Symposium on Biotechnology of Fruit Species: BIOTECHFRUIT2008 839.

[16] Aly A.A., El-Desouky W., El-Leel O.F.A. (2022). Micropropagation, Phytochemical Content and Antioxidant Activity of Gamma-Irradiated Blackberry (*Rubus fruticosus* L.) Plantlets. In Vitro Cell. Dev. Biol.-Plant.

[17] Standardi A., Micheli M. (2012). Encapsulation of In Vitro-Derived Explants: An Innovative Tool for Nurseries. Protocols for Micropropagation of Selected Economically-Important Horticultural Plants.

[18] Ahmad N., Shahid A., Javed S.B., Khan M.I., Anis M. (2015). Micropropagation of Vitex Spp. through In Vitro Manipulation: Current Status and Future Prospectives. J. Appl. Res. Med. Aromat. Plants.

[19] Rai M.K., Asthana P., Singh S.K., Jaiswal V.S., Jaiswal U. (2009). The Encapsulation Technology in Fruit Plants—A Review. Biotechnol. Adv..

[20] Bettoni J.C., Costa M.D., Souza J.A., Volk G.M., Nickel O., da Silva F.N., Kretzschmar A.A. (2018). Cryotherapy by Encapsulation-Dehydration Is Effective for In Vitro Eradication of Latent Viruses from ‘Marubakaido’ Apple Rootstock. J. Biotechnol..

[21] Wang M.-R., Bi W.-L., Bettoni J.C., Zhang D., Volk G.M., Wang Q.-C. (2022). Shoot Tip Cryotherapy for Plant Pathogen Eradication. Plant Pathol..

[22] Rihan H.Z., Kareem F., El-Mahrouk M.E., Fuller M.P. (2017). Artificial Seeds (Principle, Aspects and Applications). Agronomy.

[23] Lambardi M., Benelli C., Ozudogru E.A., Ozden-Tokatli Y. (2006). Synthetic Seed Technology in Ornamental Plants. Floriculture, Ornamental and Plant Biotechnology.

[24] Jadán M., Ruiz J., Soria N., Mihai R. (2015). Synthetic Seeds Production and the Induction of Organogenesis in Blackberry (*Rubus glaucus* Benth). Rom. Biotechnol. Lett..

[25] Gupta S., Reed B.M. (2006). Cryopreservation of Shoot Tips of Blackberry and Raspberry by Encapsulation-Dehydration and Vitrification. Cryoletters.

[26] Micheli M., Standardi A., Fernandes da Silva D. (2019). Encapsulation and Synthetic Seeds of Olive (*Olea europaea* L.): Experiences and Overview. Synthetic Seeds: Germplasm Regeneration, Preservation and Prospects.

[27] Piccioni E., Standardi A. (1995). Encapsulation of Micropropagated Buds of Six Woody Species. Plant Cell Tissue Organ Cult..

[28] Piccioni E. (1997). Plantlets from Encapsulated Micropropagated Buds of M.26 Apple Rootstock. Plant Cell Tissue Organ Cult..

[29] Gangopadhyay G., Roy S.K., Gangopadhyay S.B., Mukherjee K.K. (2009). Agrobacterium-Mediated Genetic Transformation of Pineapple Var. Queen Using a Novel Encapsulation-Based Antibiotic Selection Technique. Plant Cell Tissue Organ Cult..

[30] Germana M.A., Micheli M., Chiancone B., Macaluso L., Standardi A. (2011). Organogenesis and Encapsulation of In Vitro-Derived Propagules of Carrizo Citrange [*Citrus sinensis* (L.) Osb. × *Poncirius trifoliata* (L.) Raf]. Plant Cell Tissue Organ Cult..

[31] Naik S.K., Chand P.K. (2006). Nutrient-Alginate Encapsulation of In Vitro Nodal Segments of Pomegranate (*Punica granatum* L.) for Germplasm Distribution and Exchange. Sci. Hortic..

[32] Adriani M., Piccioni E., Standardi A. (2000). Effect of Different Treatments on the Conversion of ‘Hayward’ Kiwifruit Synthetic Seeds to Whole Plants Following Encapsulation of In Vitro-Derived Buds. N. Z. J. Crop Hortic. Sci..

[33] Sicurani M., Piccioni E., Standardi A. (2001). Micropropagation and Preparation of Synthetic Seed in M.26 Apple Rootstock I: Attempts towards Saving Labor in the Production of Adventitious Shoot Tips Suitable for Encapsulation. Plant Cell Tissue Organ Cult..

[34] Pattnaik S., Chand P.K. (2000). Morphogenic Response of the Alginate-Encapsulated Axillary Buds from In Vitro Shoot Cultures of Six Mulberries. Plant Cell Tissue Organ Cult..

[35] Gayatri M.C., Revanasiddaiah H.M. (2006). Propagation of Mulberry Variety—S54 by Synseeds of Axillary Bud. Plant Cell Tissue Organ Cult..

[36] Micheli M., Hafiz I.A., Standardi A. (2007). Encapsulation of In Vitro-Derived Explants of Olive (*Olea europaea* L. cv. Moraiolo): II. Effects of Storage on Capsule and Derived Shoots Performance. Sci. Hortic..

[37] Liberatore C.M., Rodolfi M., Beghè D., Fabbri A., Ganino T., Chiancone B. (2020). Adventitious Shoot Organogenesis and Encapsulation Technology in Hop (*Humulus lupulus* L.). Sci. Hortic..

[38] Sandoval-Yugar E.W., Dal Vesco L.L., Steinmacher D.A., Stolf E.C., Guerra M.P. (2009). Microshoots Encapsulation and Plant Conversion of *Musa* sp. cv. “Grand Naine”. Cienc. Rural.

[39] Lata H., Chandra S., Khan I.A., ElSohly M.A. (2009). Propagation through Alginate Encapsulation of Axillary Buds of *Cannabis sativa* L.—An Important Medicinal Plant. Physiol. Mol. Biol. Plants.

[40] Chand S., Singh A.K. (2004). Plant Regeneration from Encapsulated Nodal Segments of *Dalbergia sissoo* Roxb., a Timber-Yielding Leguminous Tree Species. J. Plant Physiol..

[41] Rout G.R., Das G., Samantaray S., Das P. (2001). Micropropagation of *Plumbago zeylanica* L. by Encapsulated Nodal Explants. J. Hortic. Sci. Biotechnol..

[42] Verma S.K., Rai M.K., Asthana P., Jaiswal V.S., Jaiswal U. (2010). In Vitro Plantlets from Alginate-Encapsulated Shoot Tips of *Solanum nigrum* L.. Sci. Hortic..

[43] Dave A., Joshi N., Purohit S.D. (2004). In Vitro Propagation of Chlorophytum Borivilianum Using Encapsu-Lated Shoot Buds. Eur. J. Hortic. Sci..

[44] Singh A.K., Sharma M., Varshney R., Agarwal S.S., Bansal K.C. (2006). Plant Regeneration from Alginate-Encapsulated Shoot Tips of *Phylianthus*
*amarus* Schum and Thonn, a Medicinally Important Plant Species. In Vitro Cell. Dev. Biol.—Plant.

[45] Singh S.K., Rai M.K., Asthana P., Pandey S., Jaiswal V.S., Jaiswal U. (2009). Plant Regeneration from Alginate-Encapsulated Shoot Tips of *Spilanthes acmella* (L.) Murr., a Medicinally Important and Herbal Pesticidal Plant Species. Acta Physiol. Plant.

[46] Faisal M., Anis M. (2007). Regeneration of Plants from Alginate-Encapsulated Shoots of *Tylophora indica* (Burm. f.) Merrill, an Endangered Medicinal Plant. J. Hortic. Sci. Biotechnol..

[47] Chow Y.N., Selby C., Fraser T.W., Harvey B.M.R. (1993). Basal Plate Tissue in Narcissus Bulbs and in Shoot Clump Cultures: Its Structure and Role in Organogenic Potential of Single Leaf Cultures. Ann. Bot..

[48] Stanisavljevic M. New Small Fruit Cultivars from Cacak: 1. The New Blackberry (*Rubus* sp.) Cultivar ‘Cacanska Bestrna’. Proceedings of the VII International Symposium on Rubus and Ribes 505.

[49] Himelrick D.G., Nesbitt M. Thornless Blackberry Performance on the Gulf Coast of Alabama. Proceedings of the VIII International Rubus and Ribes Symposium 585.

[50] Classic Murashige T., Skoog F. (1962). A Revised Medium for Rapid Growth and Bioassays with Tobacco Tissue Cultures. Physiol. Plant.

