# Supplementary material for: The Influence of the Explant’s Type on the Performance of Synthetic Seeds of Blackberry (Rubus spp.)
Source: Plants (Basel). 2023 Dec 21;13(1):32. doi: 10.3390/plants13010032 (PMC10781007; doi:10.3390/plants13010032)
Supplement: Supplementary file 1 [file plants-13-00032-s001.zip › plants-2621140-supplementary.pdf]

Table S1. ANOVA tables

Data Figure 1

ANOVA TABLE
Viability % for ‘Thornfree’ cultivar

EFFECT	SS	DF	MS	F	ProbF	Sign.	C.V. (%)	S.E.M.	S.E.D. 1	S.E.D. 2	Satter. DF
Propagule type	756,25	1	756,25	3,821053	0,024008	*		4,97389	7,034143		
Error Type propagule	1187,5	6	197,9167				16,43011				
Explant’s age	506,25	1	506,25	9	0,098409	ns		5,636562	3,75		
Interaction (Type x Age)	6,25	1	6,25	0,111111	0,750223	ns		5,636562	5,303301	7,971303	9,155628
Residual	337,5	6	56,25				8,759124				
Total	2793,75	15	186,25								

Propagule type x Explant's age combinations: SED 1 is for comparing means at different levels of Explant's age and same level of propagule type. SED 2 is for comparing all other means.
SED 2 has an approximate number of degrees of freedom (Satterwtwhaite approximation for degrees of freedom is reported)

ANOVA TABLE
Regeneration % for ‘Thornfree’ cultivar

EFFECT	SS	DF	MS	F	ProbF	Sign.	C.V. (%)	S.E.M.	S.E.D. 1	S.E.D. 2	Satter. DF
Propagule type	100	1	100	0,666667	0,445416	ns		4,330127	6,123724		
Error Type propagule	900	6	150				15,80316				
Explant’s age	625	1	625	3,571429	0,107679	ns		6,373774	6,614378		
Interaction (Type x Age)	25	1	25	0,142857	0,718467	ns		6,373774	9,354143	9,013878	11,92941
Residual	1050	6	175				17,06936				
Total	2700	15	180								

Propagule type x Explant's age combinations: SED 1 is for comparing means at different levels of Explant's age and same level of propagule type. SED 2 is for comparing all other means.
SED 2 has an approximate number of degrees of freedom (Satterwtwhaite approximation for degrees of freedom is reported)

Data Figure 2

ANOVA TABLE
Shoot produced (n) for ‘Thornfree’ cultivar

EFFECT	SS	DF	MS	F	ProbF	Sign.	C.V. (%)	S.E.M.	S.E.D. 1	S.E.D. 2	Satter. DF
Propagule type	35,10367	1	35,10367	213,7721	6,43E-06	**		0,14327	0,202615		
Error Type propagule	0,985264	6	0,164211				15,91726067				
Explant’s age	0,274342	1	0,274342	0,260633	0,627918	ns		0,390001	0,512981		
Interaction (Type x Age)	0,109376	1	0,109376	0,103911	0,758122	ns		0,390001	0,725464	0,551545	7,827585
Residual	6,315579	6	1,052596				15,49798041				
Total	42,78823	15	2,852548								

Propagule type x Explant's age combinations: SED 1 is for comparing means at different levels of Explant's age and same level of propagule type. SED 2 is for comparing all other means.
SED 2 has an approximate number of degrees of freedom (Satterwtwhaite approximation for degrees of freedom is reported)

ANOVA TABLE
Shoot lengh (mm) for ‘Thornfree’ cultivar

EFFECT	SS	DF	MS	F	ProbF	Sign.	C.V. (%)	S.E.M.	S.E.D. 1	S.E.D. 2	Satter. DF
Propagule type	32,418	1	32,418	21,6844	0,003479	**		0,432289	0,611349		
Error Type propagule	8,969952	6	1,494992				13,99441				
Explant’s age	4,712587	1	4,712587	1,885416	0,218824	ns		0,706619	0,790489		
Interaction (Type x Age)	0,135633	1	0,135633	0,054264	0,823546	ns		0,706619	1,117921	0,999311	11,28628
Residual	14,99696	6	2,499494				13,59511				
Total	61,23313	15	4,082209								

Propagule type x Explant's age combinations: SED 1 is for comparing means at different levels of Explant's age and same level of propagule type. SED 2 is for comparing all other means.
SED 2 has an approximate number of degrees of freedom (Satterwtwhaite approximation for degrees of freedom is reported)

ANOVA TABLE

Roots produced (n) for 'Thornfree' cultivar

EFFECT	SS	DF	MS	F	ProbF	Sign.	C.V. (%)	S.E.M.	S.E.D. 1	S.E.D. 2	Satter. DF
Propagule type	30,7438	1	30,7438	23,29961	0,002919	**		0,406125	0,574347		
Error Type propagule	7,916991	6	1,319499				16,35067				
Explant's age	0,61974	1	0,61974	0,294405	0,606961	ns		0,65427	0,725441		
Interaction (Type x Age)	0,094662	1	0,094662	0,044969	0,839082	ns		0,65427	1,025928	0,925278	11,40013
Residual	12,63034	6	2,105056				15,11343				
Total	52,00553	15	3,467035								

Propagule type x Explant's age combinations: SED 1 is for comparing means at different levels of Explant's age and same level of propagule type. SED 2 is for comparing all other means.

SED 2 has an approximate number of degrees of freedom (Satterwtwhaite approximation for degrees of freedom is reported)

ANOVA TABLE

Roots lengh (mm) for 'Thornfree' cultivar

EFFECT	SS	DF	MS	F	ProbF	Sign.	C.V. (%)	S.E.M.	S.E.D. 1	S.E.D. 2	Satter. DF
Propagule type	36,46266	1	36,46266	1,877851	0,219637	ns		1,557932	2,203249		
Error Type propagule	116,5034	6	19,41723				17,494771				
Explant's age	5,669174	1	5,669174	0,284477	0,612951	ns		2,217702	2,232062		
Interaction (Type x Age)	34,63149	1	34,63149	1,737796	0,235506	ns		2,217702	3,156612	3,136305	11,99797
Residual	119,5704	6	19,9284				13,585101				
Total	312,8371	15	20,85581								

Propagule type x Explant's age combinations: SED 1 is for comparing means at different levels of Explant's age and same level of propagule type. SED 2 is for comparing all other means.

SED 2 has an approximate number of degrees of freedom (Satterwtwhaite approximation for degrees of freedom is reported)

Data Figure 3

ANOVA TABLE

Fresh weight x explant (mg) for 'Thornfree' cultivar

EFFECT	SS	DF	MS	F	ProbF	Sign.	C.V. (%)	S.E.M.	S.E.D. 1	S.E.D. 2	Satter. DF
Propagule type	2998,42	1	2998,42	21,21573	0,003669	**		4,203124	5,944116		
Error Type propagule	847,9802	6	141,33				18,40532				
Explant's age	44,30551	1	44,30551	0,588844	0,471973	ns		5,203022	4,337092		
Interaction (Type x Age)	22,37414	1	22,37414	0,297364	0,605202	ns		5,203022	6,133575	7,358185	10,97774
Residual	451,4489	6	75,24148				17,81158				
Total	4364,529	15	290,9686								

Propagule type x Explant's age combinations: SED 1 is for comparing means at different levels of Explant's age and same level of propagule type. SED 2 is for comparing all other means.

SED 2 has an approximate number of degrees of freedom (Satterwtwhaite approximation for degrees of freedom is reported)

ANOVA TABLE

Dry weight x explant (mg) for 'Thornfree' cultivar

EFFECT	SS	DF	MS	F	ProbF	Sign.	C.V. (%)	S.E.M.	S.E.D. 1	S.E.D. 2	Satter. DF
Propagule type	26,01433	1	26,01433	72,39148	0,000144	**		0,211942	0,299732		
Error Type propagule	2,156138	6	0,359356				19,17315				
Explant's age	0,059164	1	0,059164	0,203959	0,66741	ns		0,28492	0,269294		
Interaction (Type x Age)	0,589525	1	0,589525	2,032303	0,203872	ns		0,28492	0,38084	0,402937	11,86498
Residual	1,740465	6	0,290077				19,81065				
Total	30,55962	15	2,037308								

Propagule type x Explant's age combinations: SED 1 is for comparing means at different levels of Explant's age and same level of propagule type. SED 2 is for comparing all other means.

SED 2 has an approximate number of degrees of freedom (Satterwtwhaite approximation for degrees of freedom is reported)

Data Figure 5

ANOVA TABLE

Viability % for ‘Chester’ cultivar

EFFECT	SS	DF	MS	F	ProbF	Sign.	C.V. (%)	S.E.M.	S.E.D. 1	S.E.D. 2	Satter. DF
Propagule type	625	1	625	3,571429	0,107679	ns		4,677072	6,614378		
Error Type propagule	1050	6	175				14,90564				
Explant’s age	0	1	0	0	1	ns		7,28869	7,905694		
Interaction (Type x Age)	400	1	400	1,6	0,25281	ns		7,28869	11,18034	10,30776	11,63758
Residual	1500	6	250				17,81565				
Total	3575	15	238,3333								

Propagule type x Explant's age combinations: SED 1 is for comparing means at different levels of Explant's age and same level of propagule type. SED 2 is for comparing all other means.

SED 2 has an approximate number of degrees of freedom (Satterwtwhaite approximation for degrees of freedom is reported)

ANOVA TABLE

Regeneration % for ‘Chester’ cultivar

EFFECT	SS	DF	MS	F	ProbF	Sign.	C.V. (%)	S.E.M.	S.E.D. 1	S.E.D. 2	Satter. DF
Propagule type	25	1	25	0,065934	0,80593	ns		6,884463	9,736101		
Error Type propagule	2275	6	379,1667				12,45367				
Explant’s age	0	1	0	0	1	ns		9,736101	9,736101		
Interaction (Type x Age)	25	1	25	0,065934	0,80593	ns		9,736101	13,76893	13,76893	12,0
Residual	2275	6	379,1667				12,45367				
Total	4600	15	306,6667								

Propagule type x Explant's age combinations: SED 1 is for comparing means at different levels of Explant's age and same level of propagule type. SED 2 is for comparing all other means.

SED 2 has an approximate number of degrees of freedom (Satterwtwhaite approximation for degrees of freedom is reported)

Data Figure 6

ANOVA TABLE

Shoot produced (n) for ‘Chester’ cultivar

EFFECT	SS	DF	MS	F	ProbF	Sign.	C.V. (%)	S.E.M.	S.E.D. 1	S.E.D. 2	Satter. DF
Propagule type	47,18397	1	47,18397	7,900326	0,030726	*		0,864032	1,221926		
Error Type propagule	35,83445	6	5,972408				14,37128				
Explant’s age	2,68267	1	2,68267	0,44067	0,531464	ns		1,227809	1,233664		
Interaction (Type x Age)	6,065806	1	6,065806	0,996403	0,356722	ns		1,227809	1,744664	1,736384	11,9989
Residual	36,52622	6	6,087703				15,08570				
Total	128,2931	15	8,552874								

Propagule type x Explant's age combinations: SED 1 is for comparing means at different levels of Explant's age and same level of propagule type. SED 2 is for comparing all other means.

SED 2 has an approximate number of degrees of freedom (Satterwtwhaite approximation for degrees of freedom is reported)

ANOVA TABLE

Shoot lengh (mm) for ‘Chester’ cultivar

EFFECT	SS	DF	MS	F	ProbF	Sign.	C.V. (%)	S.E.M.	S.E.D. 1	S.E.D. 2	Satter. DF
Propagule type	0,609586	1	0,609586	0,367853	0,566402	ns		0,45513	0,643651		
Error Type propagule	9,94288	6	1,657147				11,47124				
Explant’s age	0,005562	1	0,005562	0,000795	0,978425	ns		1,040253	1,322862		
Interaction (Type x Age)	5,913011	1	5,913011	0,844733	0,393495	ns		1,040253	1,870809	1,471139	8,690112
Residual	41,99913	6	6,999856				13,57623				
Total	58,47017	15	3,898012								

Propagule type x Explant's age combinations: SED 1 is for comparing means at different levels of Explant's age and same level of propagule type. SED 2 is for comparing all other means.

SED 2 has an approximate number of degrees of freedom (Satterwtwhaite approximation for degrees of freedom is reported)

ANOVA TABLE

Roots produced (n) for 'Chester' cultivar

EFFECT	SS	DF	MS	F	ProbF	Sign.	C.V. (%)	S.E.M.	S.E.D. 1	S.E.D. 2	Satter. DF
Propagule type	8,546729	1	8,546729	7,787349	0,031553	*		0,370391	0,523812		
Error Type propagule	6,585087	6	1,097514				12,80926				
Explant's age	12,032	1	12,032	9,816336	0,020245	*		0,538891	0,553559		
Interaction (Type x Age)	2,146651	1	2,146651	1,751351	0,233898	ns		0,538891	0,782851	0,762107	11,96357
Residual	7,35427	6	1,225712				14,67252				
Total	36,66473	15	2,444316								

Propagule type x Explant's age combinations: SED 1 is for comparing means at different levels of Explant's age and same level of propagule type. SED 2 is for comparing all other means.

SED 2 has an approximate number of degrees of freedom (Satterwtwhaite approximation for degrees of freedom is reported)

ANOVA TABLE

Roots lengh (mm) for 'Chester' cultivar

EFFECT	SS	DF	MS	F	ProbF	Sign.	C.V. (%)	S.E.M.	S.E.D. 1	S.E.D. 2	Satter. DF
Propagule type	3,861343	1	3,861343	0,376749	0,561882	ns		1,131874	1,600711		
Error Type propagule	61,49461	6	10,2491				17,63551				
Explant's age	186,8492	1	186,8492	46,84823	0,000478	**		1,334049	0,998548		
Interaction (Type x Age)	20,69062	1	20,69062	5,187706	0,063002	ns		1,334049	1,41216	1,88663	10,05559
Residual	23,93037	6	3,988394				18,48709				
Total	296,8261	15	19,78841								

Propagule type x Explant's age combinations: SED 1 is for comparing means at different levels of Explant's age and same level of propagule type. SED 2 is for comparing all other means.

SED 2 has an approximate number of degrees of freedom (Satterwtwhaite approximation for degrees of freedom is reported)

Data Figure 7

Fresh weight x explant (mg) for 'Chester' cultivar

EFFECT	SS	DF	MS	F	ProbF	Sign.	C.V. (%)	S.E.M.	S.E.D. 1	S.E.D. 2	Satter. DF
Propagule type	176,8287	1	176,8287	13,7988	0,009915	**		1,265642	1,789888		
Error Type propagule	76,88875	6	12,81479				17,07120				
Explant's age	22,23045	1	22,23045	5,172349	0,063295	ns		1,462563	1,036574		
Interaction (Type x Age)	1,658516	1	1,658516	0,385886	0,557317	ns		1,462563	1,465937	2,068377	9,617725
Residual	25,78764	6	4,29794				17,83937				
Total	303,394	15	20,22627								

Propagule type x Explant's age combinations: SED 1 is for comparing means at different levels of Explant's age and same level of propagule type. SED 2 is for comparing all other means.

SED 2 has an approximate number of degrees of freedom (Satterwtwhaite approximation for degrees of freedom is reported)

ANOVA TABLE

Dry weight x explant (mg) for 'Chester' cultivar

EFFECT	SS	DF	MS	F	ProbF	Sign.	C.V. (%)	S.E.M.	S.E.D. 1	S.E.D. 2	Satter. DF
Propagule type	2,399057	1	2,399057	15,68973	0,007443	**		0,138251	0,195516		
Error Type propagule	0,917437	6	0,152906				16,38449				
Explant's age	0,33769	1	0,33769	3,282716	0,119971	ns		0,178807	0,160366		
Interaction (Type x Age)	0,095412	1	0,095412	0,927512	0,372702	ns		0,178807	0,226792	0,252871	11,55768
Residual	0,617215	6	0,102869				19,83326				
Total	4,366811	15	0,291121								

Propagule type x Explant's age combinations: SED 1 is for comparing means at different levels of Explant's age and same level of propagule type. SED 2 is for comparing all other means.

SED 2 has an approximate number of degrees of freedom (Satterwtwhaite approximation for degrees of freedom is reported)
